# Peer Review of “Towards Evaluating the Diagnostic Ability of LLMs (Preprint)”

**DOI:** 10.2196/69830

**Published:** 2024-12-17

**Authors:** Daniela Saderi, Randa Salah Gomaa Mahmoud, Goktug Bender, Olajumoke Ope Oladoyin, Paul Hassan Ilegbusi, Arya Rahgozar, Manikant Roy, Maria Machado, Benjamin Senst, Clara Amaka Nkpoikanke Akpan, Nour Shaballout, Sylvester Sakilay, Sandeep Vohra, Mitchell Collier, Morufu Olalekan Raimi, Uday Kumar Chalwadi

**Affiliations:** 1 PREreview, Portland, OR, United States, 1 1234567789; 2 Faculty of Human Medicine, Zagazig University, Zagazig, Egypt; 3 McGill University, Montreal, QC, Canada; 4 UTHealth Houston, Houston, TX, United States; 5 Ondo State College of Health Technology, Akure, Nigeria; 6 Ottawa Hospital Research Institute, Ottawa, ON, Canada; 7 IIT Delhi, Delhi, India; 8 Storytelling for Science, Portugal; 9 Medizinische Hochschule, Brandenburg, Germany; 10 Michael Okpara University of Agriculture, Umudike, Nigeria; 11 Medizinische Hochschule Hannover, Hannover, Germany; 12 Federal University, Otuoke, Nigeria; 13 Louisiana State University Health Sciences Center Shreveport, Shreveport, LA, United States

**Keywords:** generative AI, LLM, GPT-4, RAG, clinical medicine, diagnosis, retrieval-augmented generation, large language model, artificial intelligence

*This is a peer-review report submitted for the preprint “Towards Evaluating the Diagnostic Ability of LLMs.”*

This review is the result of a virtual collaborative live review discussion organized and hosted by PREreview and JMIR Publications on November 14, 2024. The discussion was joined by 29 people: 2 facilitators, 2 members of the JMIR Publications team, 2 preprint authors, and 23 live review participants, including 2 who agreed to be named here but did not contribute to compiling this report: Junaidu Abubakar and Hafsat Ahmad. The authors of this review have dedicated additional asynchronous time over the course of 2 weeks to help compose this final report using the notes from the live review. We thank all participants who contributed to the discussion and made it possible for us to provide feedback on this preprint.

## Summary

The study [1] was designed to elucidate the predictive ability of artificial intelligence–assisted tools such as large language models (LLMs) and to explore the potential of these models to accurately predict diagnostic codes. The study also tried to investigate if retrieval-augmented generation (RAG) could be an adjuvant tool to enhance diagnostic accuracy. By evaluating the models’ diagnostic performance, the study aims to explore artificial intelligence’s potential to reduce cognitive diagnostic errors.

To address the research questions, the authors compared the diagnostic performance of 9 different LLMs from 6 different companies, using a standard patient dataset and diagnostic codes. The authors randomly selected 1000 patients’ clinical data from the MIMIC-IV database, while their corresponding diagnosis codes in the billing records were treated as ground truth. LLMs were used to generate diagnoses using doctor-engineered prompts with one-shot learning. The results of LLM-generated diagnoses were compared with those of the ground truth (information gathered from billing reports). The possible evaluation outcomes were hit, noninferable, and missed. It was concluded that LLMs could be a viable avenue for application in diagnostic efforts. Among the models used, the GPT-4.0 and Claude Sonnet 3.5 showed the highest hit rates. Moreover, RAG further improved the hit rate of GPT-4.0.

The authors sought to address the issue of cognitive diagnostic errors in clinical practice by studying the diagnostic accuracy of a range of LLMs in this context. They also tested the ability of the recent RAG framework to enhance the performance of LLMs. This study paved the way for future research to see how these models would catch up with the complex and sensitive context of medical diagnostics. The authors’ transparency about limitations mirrors research honesty, establishing a strong base for future studies. However, there are some notable weaknesses in the study, such as the limited data sample, the noninclusion of some clinical data from the analysis, and the study did not assess model prediction biases recommending future study and enhancement.

Below, we list the concerns that were raised during the live review event on November 14, 2024, by participants and further elaborated on by the authors of this review to be shared openly on this platform.

## Major Concerns and Suggested Improvements

### Title Revision

- The current title does not fully capture the scope of the study; hence, it needs to be reconsidered. It would be nice if abbreviations were avoided in the title. Therefore, we recommend the authors of the study change “LLM“ to “Large Language Models” in the title.

### Abstract and Introduction Clarity

- The abstract and the introduction lack a clear statement of the study’s aim. It is therefore expedient to revise the abstract to include the objectives, methodology, key results, and conclusion. The Introduction should have a clear research aim.
- Note: During the call, the authors shared that a revised version of the abstract had been generated and shared so it is possible that the latest version has addressed this concern. We invite the authors to share their updated version in the comment section of this review.

### Physician Comparison With LLMs

- The study does not explore how diagnoses differ from physicians using only the data provided to the LLM. It would be advisable to include a comparative analysis to evaluate diagnosis accuracy and the prioritization of additional tests between physicians and LLMs.
- Furthermore, the absence of actual data around patient history and other diagnostic parameters beyond what was reported in billing reports (reported as “ground truth” in the study) is a weakness. This can lead to an incomplete or partial diagnosis being labeled as the final diagnosis, leading to miscalculations about the accuracy of LLMs.

### Model Selection Rationale and Evaluation Metrics

- The Method section is limited in its description of the methodology used in the study. It would be helpful to include more information on the rationale for the model selection and describe differences between GPT-4 variants to help readers understand the comparative approach.
- Furthermore, the choice of “hit rate” as the primary evaluation metric is unclear, and its limitations are not discussed in sufficient detail. It would be helpful if the choice of hit rate over other metrics (eg, precision or *F*_1_-score) as well as the limitations the hit rate may introduce were discussed more thoroughly.

### Methodology and RAG Integration Details

- The role of RAG in the diagnostic process, including how relevant information was retrieved and implemented to enhance the diagnostic process and performance needs to be elaborated further as it constitutes a novel part of the study. This issue was highlighted as one of the particular concerns, as without more details, many questions remain unanswered and that could compromise the credibility of the study.

### Data Interpretation and Population-Specific Reference Ranges

- Reference ranges used for diagnoses are not adequately explained. The authors are encouraged to clarify if the reference ranges are population-specific or if they align with the dataset characteristics.
- In general, reviewers suggest authors add more details about the nature of the data beyond referring to them as “test results” in the manuscript. For example, it would be helpful to know more about the meaning and interpretation of the homogeneity of the test results and the implications of it on the evaluation of the method.
- Provide a statistical analysis to demonstrate that the differences in diagnostic hit rates for the LLMs are statistically significant in the range of 98.5-99.8.

### Discussion

- It would be helpful to discuss why GPT-4.0 and Claude 3.5 Sonnet performed better than others, potentially due to architectural differences or data training sources.
- It would also be important to discuss why specific diagnoses (eg, diabetes) were among the best hits and most frequent misses.

### Limitations of Study Design

- The limitations could be explicitly outlined in a separate section of the Discussion for transparency and clarity. For example, the authors may include a discussion around the fact that the sample size (1000 patients) may be too small to generalize the findings, potential issues related to relying on billing reports as ground truth, and considerations of hallucinations or failure scenarios of LLMs in real-world settings. In such a section, the authors may also explore ideas related to using larger and more diverse datasets in similar future research.

### Figures and Tables

- The figures and tables in the study lack clarity and, at times, key information (eg, patient demographics, disease types are missing). The authors are advised to add clarity to the data visualizations, label axes, and include interpretive analyses to all figures, but in particular for Tables 1 and 2, and Figure 2. They are also advised to discuss specific trends such as frequent misses for certain conditions.

### Reproducibility

- The reproducibility of the study is hindered by the lack of clear documentation on LLM settings, dataset transformations, and code. It is suggested that the authors provide the full details of the LLM configurations, processing steps, and code availability.
- For example, it would be helpful to know the rationale for limiting the LLM output tokens to 4096. How could this be relevant to the “human” diagnostic process? Were some predictions judged “more likely” than others?

### Bias and Real-World Application

- Potential biases in LLM predictions and challenges in clinical adoption are not addressed in the study. It is advised that the authors add a section on potential biases and practical integration challenges. They need to include future work on improving model robustness and fairness.

## Minor Concerns and Suggested Improvements

### Abbreviation Usage

- Key abbreviations (eg, LLM, RAG) were not defined at first use. The authors are encouraged to define all abbreviations when first indicated in the abstract and body of the study (eg, “electronic health records (EHRs)” when first mentioned, then “EHR” at later mentions).

### Language

- There are several typos and some grammatical errors, incomplete sentences, and contractions that reduce the readability of the study; hence, the authors are encouraged to consider thorough proofreading and editing to improve the reading experience and interpretation of the study. This is a minor concern that may be well addressed by the copyeditors of the journal that will publish the manuscript.

### Ethical Statement Clarity

- The ethical considerations for using MIMIC-IV data are not explicitly referenced. The authors should state that the dataset is deidentified and describe access restrictions for researchers. Some reviewers had concerns about the need for ethical approval given the use of patient data, but others reported that ethical approval may not be needed given the public nature of the data used.
- Furthermore, it would be helpful to add a discussion around the potential risk of bias introduced by LLMs and its large implications on diagnosis and the field of medicine at large.

### False-Positive and False-Negative Rates

- The explanation of false-positive and false-negative rates in the study is inadequate; hence, the authors are invited to include specific examples and explanations of why certain diagnoses were misclassified.

### Conclusions

- The author should consider adding a section that examines potential biases in LLM predictions and the practical challenges of using these models in hospital settings. Furthermore, it would be helpful to further highlight practical takeaways or future directions, emphasizing actionable insights and specific areas for future research (eg, integrating multimodal data sources or fine-tuning models with diverse clinically annotated datasets).

### Comparative Model Performance

- Performance differences between models in the study are not sufficiently elaborated on in the Discussion. The authors are invited to explore why certain models performed better, considering architectural differences and training data sources.
- Reviewers also advised authors to consider human vetting for the evaluation to provide an additional layer of confidence to get the experts to reflect on the LLM answers and explanations.

### Hyperbolic Language

- Words like “stunning” are overly subjective. The use of neutral language is advised in the manuscript, and the authors are invited to justify claims with supporting data.

### Dataset Limitations

- Rare diseases may not be adequately represented in the study. The authors should address how dataset limitations affect diagnostic performance and include rare disease cases in future studies.

### Citations to Methods and Tools

- Where possible, add citations to specific LLM and RAG tools used, such as technical references from Google, OpenAI, etc, to aid readers in finding more information on these tools.
- Authors are advised to complete their statements instead of just including a citation. For example, “In this case, the further tests the LLM is instructed to suggest [2] are of crucial importance to understand exact disease pathology.”
- Provide an explanation of the sentence “NEJM Case Challenges are notoriously hard” and provide a reference. Potentially, reconsider the use of the extreme adverb “notoriously”—perhaps “well known to be.”

### Presentation of Methods

- For readability, reformat the list of LLMs used into a table with separate columns for name, version, and settings.

## Abbreviations

LLM: large language model
RAG: retrieval-augmented generation
